# Mentalization, attachment, and subjective identity

**DOI:** 10.3389/fpsyg.2015.01022

**Published:** 2015-07-21

**Authors:** Rossella Guerini, Massimo Marraffa, Claudio Paloscia

**Affiliations:** ^1^Center for Mind/Brain Sciences, University of TrentoRovereto, Italy; ^2^Department of Philosophy, Communication and Media Studies, Roma Tre UniversityRome, Italy; ^3^Department of Psychology, Niccolò Cusano UniversityRome, Italy

**Keywords:** attachment, borderline personality disorder, introspection, mindreading, subjective identity

In a life-span perspective, Baglio and Marchetti make the hypothesis of “the existence of multiple kinds of Theory of Mind” and urge the transition from a discrete to a dimensional approach in the study of mentalization (“ToM may vary along a quantitative and a qualitative continuum”). We resist such a plea and argue that we can stick to a discrete approach which posits just a single early-developing mindreading system, and then works out a “third-person first” perspective on mentalization, according to which the understanding of other minds both ontogenetically precedes and grounds the understanding of our own minds. In this third-person first framework, Baglio and Marchetti's claim that mentalization is “a multifaceted set of competences liable to influence—and be influenced by—a manifold of psychosocial aspects” is reformulated as follows: first-person mentalization evolves in an interplay of third-person mentalization, autobiographical memory and socio-communicative skills attuned by cultural variables. Let us examine these points one by one.

In the first place, we take a *nativist-modularist* perspective on third-person mentalization (henceforth “mindreading”). After Onishi and Baillargeon's (2005) groundbreaking paper, enough evidence has accumulated to endorse the hypothesis that a form of primary mindreading is not a developmental achievement, but an innate social-cognitive evolutionary adaptation (Baillargeon et al., 2013, 2014). Such adaptation is implemented by a mechanism computing a body of knowledge specific to the domain of naïve psychology, which comes online during the first year of age (Carruthers, 2013). Such early-developing domain-specific mindreading mechanism underpins *basic* mentalistic abilities (including the metarepresentational capacity to pass spontaneous-response false belief tests) which are to be distinguished from the full range of mature activities that may employ such skills. During the development these basic mindreading abilities “may eventually be recruited by higher cognitive processes for more complex tasks,” thus *higher-order mentalistic activities* may emerge, which “may well interact (in a non-modular way) with other cognitive processes, and may not be uniform across individuals or cultures” (Scholl and Leslie, 1999, p. 140). The focus on the neurocognitive bases of mentalizing abilities, therefore, does not rule out the influence of socio-cultural variables.

In the second place, we take a *cognitive-constructivist* stance on the debate on first-person mentalization (henceforth “introspection”). The argument for this claim builds on Carruthers' (2011) Interpretive Sensory-Access (ISA) account of the nature and sources of self-knowledge, which is a strong case for the claim that mindreading has a *functional* and *evolutionary* priority over introspection. According to the ISA account, although we can have non-interpretive access to our own sensory and affective states, the self-attribution of propositional attitude states is always a process of self-interpretation that exploits the same sensory channels that we utilize when working out other people's mental states.

Carruthers's theory of introspective self-knowledge, however, does not predict that mindreading is also *developmentally* prior to introspection (Carruthers, 2009b, p. 167). By contrast, we make just such a claim.

In an attempt to explain why we have the (false) intuition that there is introspection for our thoughts, Carruthers takes very seriously Wilson's (2002) hypothesis that the self-transparency assumption “may make it easier for subjects to engage in various kinds of adaptive self-deception, helping them build and maintain a positive self-image” (Carruthers, 2009a, p. 138, n. 5). Moreover, in looking at the possibility that the emergence of introspection is a by-product of the evolution of mindreading, Carruthers considers such possibility as compatible with the hypothesis that introspection “might have come under secondary selection thereafter, perhaps by virtue of helping to build and maintain a positive self-image, as Wilson […] suggests” (Carruthers, 2009a, p. 128).

Thus, here Carruthers is opening the door to the topic of defense mechanisms. Moreover, the ISA theory heavily draws on the confabulation data from the huge cognitive dissonance and causal attribution literatures (2011, chap. 11), and such data—we believe—can hardly be separated from the topic of the construction and maintenance of “a positive self-image.” There is a problem, though. Carruthers' (2011) focus is on introspection construed as knowledge of one's own current mental states; and this knowledge “is arguably more fundamental than knowledge of oneself as *a self with an ongoing mental life*” (Carruthers et al., 2012, p. 14; italics added). Now, insofar as Carruthers takes introspection merely as a competence to self-attribute one's own current mental states, Wilson's hypothesis of the self-defensive nature of introspection cannot be built into the ISA theory. For the topic of defenses makes sense only in the context of the construction and protection of the psychological self-consciousness or subjective identity (“a self with an ongoing mental life”). But once introspection is seen in this context it becomes possible to make the hypothesis that it *develops* through the act of turning on oneself the competence to mind-read others; and that this occurs through that socio-communicative interaction with caregivers (and successively other social partners) investigated in attachment theory research [this seems to be what is at stake in Fernyhough's (2009) and Hernik et al.'s (2009) commentaries on Carruthers (2009a)].

As regards attachment theory, it is to be noticed that it builds within a contextualist and systemic framework, where (individual) biology and (social) relationality go hand in hand. Individuals are pre-wired to the interpersonal relationship from the birth, and mindreading is integral to such pre-organization. It makes perfect sense, therefore, that the early-developing core mindreading system is an innate social-cognitive adaptation that is independent of the attachment system (Gergely and Unoka, 2008). This leads us to reject the hypothesis, variously put forward by a number of attachment theorists and infant researchers, that there is an inherent causal and functional link between the quality of early infant attachment on the one hand, and the development of mindreading on the other.

When we take into consideration introspection, in contrast, the child's socio-communicative interaction with caregivers is *constitutively* involved in the construction of the virtual inner space of the mind—an introspective (as opposed to bodily) form of self-consciousness that then evolves as narrative identity. The development of introspection is therefore an outward-in process through which a subject constructs itself as *psychologically* self-conscious (and not only as *physically* self-conscious) in an interplay of metarepresentational abilities, autobiographical memory, and socio-communicative capacities modulated by socio-cultural variables. The young child who turned her mindreading abilities upon herself under the thrust of caregivers' mind-minded talk, by the end of the preschool years begins to grasp her introspective self-description as rationalized in terms of autobiography (Fivush, 2011).

This process of narrative self-construction is inseparably cognitive and affective in nature. Attachment theory and infant research have shown that affective growth and construction of identity cannot be separated. The description of the self that from 2 to 3 years of age the child keenly pursues is an “accepting description,” i.e., a description that is indissolubly cognitive (as *definition* of self) and emotional-affectional (as *acceptance* of self). Briefly, the child needs a clear and consistent capacity to describe itself, fully legitimized by the caregiver and socially valid. Accordingly, one cannot ascribe concreteness and solidity to one's own self-consciousness if the latter does not possess, at its core, a description of identity that must be clear and, indissolubly, “good” as worthy of being loved.

The incessant construction and reconstruction of an acceptable and adaptively functioning identity is therefore the process through which our intra- and inter-personal balances are produced, and hence a foundation of psychological well-being and mental health. This finds illustration in the developmental psychopathology of attachment, according to which the abusive or seriously neglective behaviors of the attachment figures may give rise to disturbances in identity.

“Identity” is a central diagnostic criterion for personality disorders in DSM-5. Here we will look briefly at Borderline Personality Disorder (BPD), this being a clinical condition that—as Hernik et al. (2009) suggest—“may become an important source of new data that could illuminate relationships between mindreading and self-awareness and their developmental antecedents” (p. 148).

In the first place, the majority of the studies that investigated the ability of BPD patients to mind-read other people found an equal or superior mindreading ability in BPD. In Franzen et al.'s (2011) economic exchange game experiment, for example, patients with BPD had to recognize fair or unfair attitudes and deception through emotional cues from facial expressions of others, attributing mental states in the interaction with social partners. They turned out to perform better than healthy controls. This makes perfect sense when mindreading is analyzed in the context of the attachment history of the patients with BPD. In Franzen et al.'s (2011) virtual trust game participants must recognize others' intentions in a situation in which the other person could not be cooperative and/or could potentially hurt. BPD patients have been highly trained to mind-read, being their relationship with caregivers characterized by the need to protect themselves by means of an anticipation of neglectful or abusive behaviors, learning to read signals of threat or rejection (Linehan, 1995).

Second, BPD patients' impairments in mentalization cannot be conceptualized independently from their difficulties with autobiographical memory. Let us consider Preißler et al.'s (2010) study, where patients with BPD were asked to perform two higher-order mentalization tasks—Baron-Cohen et al.'s (2001) “Reading the Mind in the Eyes” (RME) task; and the “Movie for the Assessment of Social Cognition” (MASC: Dziobek et al., 2006), a more ecologically valid task in which participants are required to attribute mental states to movie characters in an everyday life-relevant context. BPD patients' performance on the RME did not show any impairments in mentalization compared to non-clinical controls. In the MASC, in contrast, PTSD intrusive symptomatology in the BPD group played an important role in the emergence of disturbed recognition of the feelings, thoughts, and intentions of the movie characters.

This is well in line with Dziobek et al.'s (2011) study, which used an fMRI adaptation of the Multifaceted Empathy Test. A region comprising the left superior temporal sulcus and gyrus (STS/STG) seems to play a role in higher-order mentalizing tasks such as those mentioned above (see, e.g., Saxe and Kanwisher, 2003). Now, Dziobek et al. found a reduced activation of the STS/STG region in BPD patients who reported high levels of recurring traumatic memories.

This leads us to a third point. The caregiver's neglect of a child's emotional responses is assumed to play a central role in the etiology of BPD (Lyons-Ruth et al., 2005). On the one hand, the absence of empathic affect-regulative-mirroring interactions may prevent the child from creating “the necessary mappings between the emerging causal representations of emotional states in others and emerging distinct emotional self-states,” which in turn may give rise to “compromised second-order representations of self-states” (Hernik et al., 2009, p. 148). On the other hand, BPD individuals' need to implement strategies to cope with the deficit in affect self-regulation may contribute to the disruption of autobiographical memory (see, e.g., Van den Broeck et al., 2012, 2015). In BPD patients, then, an interplay of impairments in first-person mentalization and autobiographical memory occurs, which disrupts the development of “autobiographical reasoning.” This is the ability to create relations between different parts of one's past, present, and future life and one's personality and development; by so doing, it embeds personal memories in a culturally, temporally, causally, and thematically coherent life story, thus offering ways of establishing and re-establishing the diachronic continuity of the self (Habermas, 2011). The impairment of autobiographical reasoning, therefore, leads to identity disturbance—a “markedly and persistently unstable self-image or sense of self” (APA, 2013, p. 664)—which has long been considered one of the defining features of BPD (see, e.g., Adler et al., 2012; Jørgensen et al., 2012).

In summary, we maintained that first-person mentalization/introspection is not simply a matter of self-attributing one's own current mental states; it is rather a higher-order mentalistic activity that gives shape to a psychological (as opposed to bodily) form of self-consciousness. Furthermore, introspection cannot be singled out from the context of that construction and defense of subjective identity that is the key to the developmental psychopathology of attachment. This was illustrated by the inextricable interweaving of mentalization, autobiographical reasoning, and identity disturbance in BPD patients.

## Conflict of interest statement

The authors declare that the research was conducted in the absence of any commercial or financial relationships that could be construed as a potential conflict of interest.

## References

[B1] Adler J. M. Chin E. D. Kolisetty A. P. Oltmanns T. F. (2012). The distinguishing characteristics of narrative identity in adults with features of Borderline Personality Disorder: an empirical investigation. J. Pers. Disord. 26, 498–512. 10.1521/pedi.2012.26.4.498 22867502 PMC3434277

[B2] APA (American Psychiatric Association) (2013). Diagnostic and Statistical Manual of Mental Disorders, 5th Edn. Washington, DC: American Psychiatric Association.

[B3] Baillargeon R. He Z. Setoh P. Scott R. M. Sloane S. Yang D. Y.-J. (2013). False-belief understanding and why it matters: the social-acting hypothesis, in Navigating the Social World, eds Banaji M. R. Gelman S. A. (New York, NY: Oxford University Press), 88–95.

[B4] Baillargeon R. Setoh P. Sloane S. Jin K. Bian L. (2014). Infant social cognition: psychological and sociomoral reasoning, in The Cognitive Neurosciences, eds Gazzaniga M. S. Mangun G. R. (Cambridge, MA: MIT Press), 7–14.

[B5] Baron-Cohen S. Wheelwright S. Hill J. Raste Y. Plumb I. (2001). The “reading the mind in the eyes” test revised version: a study with normal adults, and adults with Asperger syndrome or high-functioning autism. J. Child Psychol. Psychiatry 42, 241–251. 10.1111/1469-7610.00715 11280420

[B6] Carruthers P. (2009a). How we know our own minds: the relationship between mindreading and metacognition. Behav. Brain Sci. 32, 121–138. 10.1017/S0140525X09000545 19386144

[B7] Carruthers P. (2009b). Mindreading underlies metacognition. Behav. Brain Sci. 32, 164–176. 10.1017/S0140525X09000831 19386144

[B8] Carruthers P. (2011). The Opacity of Mind: The Cognitive Science of Self-Knowledge. Oxford: Oxford University Press.

[B9] Carruthers P. (2013). Mindreading in infancy. Mind Lang. 28, 141–172. 10.1111/mila.1201424646207

[B10] Carruthers P. Fletcher L. Ritchie B. (2012). The evolution of self-knowledge. Philos. Topics 15, 13–37. 10.5840/philtopics201240212

[B11] Dziobek I. Fleck S. Kalbe E. Rogers K. Hassenstab J. Brand M.. (2006). Introducing MASC: A Movie for the Assessment of Social Cognition. J. Autism Dev. Disord. 36, 623–636. 10.1007/s10803-006-0107-0 16755332

[B12] Dziobek I. Preissler S. Grozdanovic Z. Heuser I. Heekeren H. R. Roepke S. (2011). Neuronal correlates of altered empathy, and social cognition in borderline personality disorder. Neuroimage 15, 539–548. 10.1016/j.neuroimage.2011.05.005 21586330

[B13] Fernyhough C. (2009). What can we say about the inner experience of the young child? Behav. Brain Sci. 32, 143–144. 10.1017/S0140525X0900061215117389

[B14] Fivush R. (2011). The development of autobiographical memory. Annu. Rev. Psychol. 62, 559–582. 10.1146/annurev.psych.121208.131702 20636128

[B15] Franzen N. Hagenhoff M. Baer N. Schmidt A. Mier D. Sammer G.. (2011). Superior “theory of mind” in borderline personality disorder: an analysis of interaction behavior in a virtual trust game. Psychiatry Res. 15, 224–233. 10.1016/j.psychres.2010.11.012 21129781

[B16] Gergely G. Unoka Z. (2008). Attachment, affect-regulation and mentalization, in Mind to Mind: Infant Research, Neuroscience and Psychoanalysis, eds Jurist E. L. Slade A. Bergner S. (New York, NY: Other Press), 50–87.

[B17] Habermas T. (2011). Autobiographical reasoning: arguing and narrating from a biographical perspective. New Dir. Child Adolesc. Dev. 131, 1–17. 10.1002/cd.285 21387528

[B18] Hernik M. Fearon P. Fonagy P. (2009). There must be more to development of mindreading and metacognition than passing false belief tasks. Behav. Brain Sci. 32, 147–148. 10.1017/S0140525X0900065X

[B19] Jørgensen C. R. Berntsen D. Bech M. Kjølbye M. Bennedsen B. E. Ramsgaard S. B. (2012). Identity-related autobiographical memories and cultural life scripts in patients with Borderline Personality Disorder. Conscious. Cogn. 21, 788–798. 10.1016/j.concog.2012.01.010 22356875

[B20] Linehan M. M. (1995). Understanding Borderline Personality Disorder. New York, NY: Guilford Press.

[B21] Lyons-Ruth K. Yellin C. Melnick S. Atwood G. (2005). Expanding the concept of unresolved mental states: hostile/helpless states of mind on the Adult Attachment Interview are associated with disrupted mother-infant communication and infant disorganization. Dev. Psychopathol. 17, 1–23. 10.1017/S0954579405050017 15971757 PMC1857275

[B22] Onishi K. H. Baillargeon R. (2005). Do 15-month-old infants understand false beliefs? Science 308, 255–258. 10.1126/science.1107621 15821091 PMC3357322

[B23] Preißler S. Dziobek I. Ritter K. Heekeren H. R. Roepke S. (2010). Social cognition in Borderline Personality Disorder: evidence for disturbed recognition of the emotions, thoughts, and intentions of others. Front. Behav. Neurosci. 4:182. 10.3389/fnbeh.2010.00182 21151817 PMC2999836

[B24] Saxe R. Kanwisher N. (2003). People thinking about people: the role of the temporo-parietal junction in “theory of mind.” Neuroimage 19, 1835–1842. 10.1016/S1053-8119(03)00230-1 12948738

[B25] Scholl B. J. Leslie A. M. (1999). Modularity, development and “Theory of mind”. Mind Lang. 14, 131–153. 10.1111/1468-0017.00106

[B26] Van den Broeck K. Claes L. Pieters G. Raes F. (2012). Memory specificity in borderline personality disorder: associations with depression and self-discrepancy. J. Behav. Ther. Exp. Psychiatry 43, S51eS59. 10.1016/j.jbtep.2011.02.001 23200432

[B27] Van den Broeck K. Claes L. Pieters G. Hermans D. Raes F. (2015). Overgeneral memory in borderline personality disorder, in Clinical Perspectives on Autobiographical Memory, eds Watson L. A. Berntsen D. (Cambridge: Cambridge University Press), 221–241.

[B28] Wilson T. (2002). Strangers to Ourselves. Cambridge, MA: Harvard University Press.

